# Comparison of Electric- and Magnetic-Cardiograms Produced by Myocardial Ischemia in Models of the Human Ventricle and Torso

**DOI:** 10.1371/journal.pone.0160999

**Published:** 2016-08-24

**Authors:** Erick A. Perez Alday, Haibo Ni, Chen Zhang, Michael A. Colman, Zizhao Gan, Henggui Zhang

**Affiliations:** 1 Biological Physics Group, School of Physics and Astronomy, University of Manchester, Manchester, United Kingdom; 2 Applied superconductivity Research Center, School of Physics, Peking University, Beijing, China; 3 Theoretical Physics Group, School of Physics and Astronomy, University of Manchester, Manchester, United Kingdom; Indiana University, UNITED STATES

## Abstract

Myocardial ventricular ischemia arises from a lack of blood supply to the heart, which may cause abnormal repolarization and excitation wave conduction patterns in the tissue, leading to cardiac arrhythmias and even sudden death. Current diagnosis of cardiac ischemia by the 12-lead electrocardiogram (ECG) has limitations as they are insensitive in many cases and may show unnoticeable differences to normal patterns. As the magnetic field provides extra information on cardiac excitation and is more sensitive to tangential currents to the surface of the chest, whereas the electric field is more sensitive to flux currents, it has been hypothesized that the magnetocardiogram (MCG) may provide a complementary method to the ECG in ischemic diagnosis. However, it is unclear yet about the differences in sensitivity regions of body surface ECG and MCG signals to ischemic conditions. The aim of this study was to investigate such differences by using 12-, 36- ECG and 36-MCG computed from multi-scale biophysically detailed computational models of the human ventricles and torso in both control and ischemic conditions. It was shown that ischemia produced changes in the ECG and MCG signals in the QRS complex, T-wave and ST-segment, with greater relative differences seen in the 36-lead ECG and MCG as compared to the 12-leads ECG (34% and 37% vs 26%, respectively). The 36-lead ECG showed more averaged sensitivity than the MCG in the change of T-wave due to ischemia (37% vs 32%, respectively), whereas the MCG showed greater sensitivity than the ECG in the change of the ST-segment (50% vs 40%, respectively). In addition, both MCG and ECG showed regional-dependent changes to ischemia, but with MCG showing a stronger correlation between ischemic region in the heart. In conclusion, MCG shows more sensitivity than ECG in response to ischemia, which may provide an alternative method for the diagnosis of ischemia.

## Introduction

Ischemic heart disease is one of the leading causes of death in developed countries and worldwide [1–3]. Coronary artery occlusion can cause, within hours, cell death in ischemic myocardium [1]. This results from a lack of blood flow to the heart which decreases partially or completely the oxygen supply to the cell, damaging the muscle [1]. Significant ischemic regions within the heart can promote abnormal excitation wave conduction and repolarization patterns, leading to ventricular arrhythmias and even sudden cardiac death [4,5]. Therefore, being able to detect, quantify and locate the site of acute transient ischemic regions in the heart by non-invasive techniques is a clinically important challenge [3,6].

The 12-lead electrocardiogram (ECG) has been implemented as a standard bedside evaluation procedure for cardiac condition diagnosis for multiple decades [3,7]. Unfortunately, the standard 12-lead ECG has been shown to be insensitive to cardiac ischemia; the ECG waveforms of patients with ischemia may only differ by 15–30% compared to none-ischemic patients [3,4,6,8]. This suggests that the 12-lead ECG provides insufficient information for satisfactory diagnosis of ischemia. Other non-invasive techniques, including radionuclide methods [9], magnetic resonance imaging [10] and positron computed tomography [11], are far more sensitive to the detection of ischemia. However, they are highly expensive and time consuming, and therefore not practical for day-to-day, bedside monitoring and detection of silent ischemia (i.e. asymptomatic ischemia which does not present as an arrhythmia) [12–14].

Previous studies have shown that multi-lead ECG configurations provide more information for the diagnosis of irregular cardiac conduction and repolarization patterns than the standard 12-lead ECG [8,12,15]. Moreover, the magnetic field produced by the electrical activity of the heart may provide a greater level of detail of cardiac excitation compared to the body surface potential (BSP), because magnetocardiograms (MCG) are more sensitive to currents tangential to the surface of the chest than ECGs. Combined with its high independence to inhomogeneities in electrical resistivity inside the tissues of the body and on the skin [12,16,17], the MCG therefore provides a potential practical alternative to the ECG for monitoring the cardiac conditions. However, detailed correlation between the presence of ischemia and the characteristics of the MCG has yet to be established.

In this study, we aim to compare and quantify the effects of the presence of ventricular ischemia on BSP and MCG maps and the 36-lead ECG and MCG recordings derived from these maps, in order to compare the most sensitive regions of the body related to the presence of ischemia. This was achieved through application of a multi-scale computational model of the human ventricles to simulate the effects of ischemic zones on electrical wave propagation throughout the heart. Then, using the simulation data of the human ventricles, the electric and magnetic forward problems were solved in a torso model to obtain the BSP and MCG maps, respectively.

## Methods

### Experimental ECG and MCG equipment and data acquisition

A self-developed four-channel high temperature ratio-frequency superconducting quantum interference device (HTc-rf-SQUID) Bio-magnetometer (Peking University, China) [18] was used to detect the cardiac magnetic field. The multichannel system (36-lead MCG) was arranged in a squared structure and was placed on the front of the chest and recorded the vertical component of the MCG signal, which is, the normal component of the magnetic field to the chest surface of the subject [18]. The sensor array covered a square area of 80x80 mm^2^, and the distance between adjacent channels was 40 mm. The system was operated inside a magnetically shielded room after inserting the system in a resin rod of identical epoxy-reinforced nonmagnetic glass fiber crystals containing liquid helium [18]. The noise spectral density of the fabricated magnetometers was less than 100fT/√Hz in the white noise region [18,19]. A 36-lead ECG was also obtained placing the electrodes on the surface of the body, in a similar position of the MCG sensors, in order to compare the measurements [18]. The MCG and ECG experimental data was obtained from a 25 years old healthy (no cardiac disease presented) subject.

### Description of mathematical models

**Fig 1:**
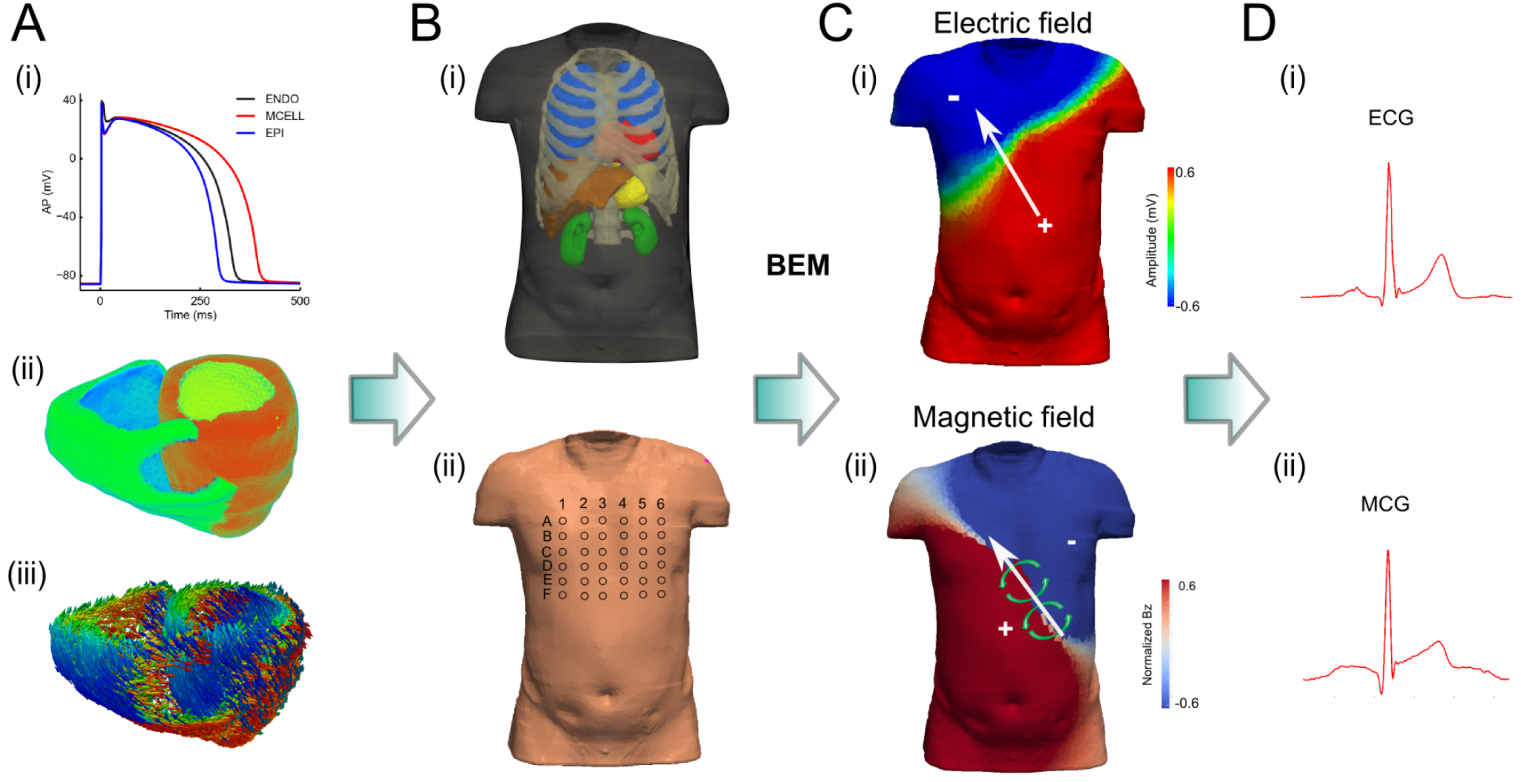
Multi-scale computer models of the human ventricles and torso. (A) Computational model of the human ventricles showing (i) AP, (ii) anatomically accurate structure, and (iii) myofibre orientations derived from DTMRI imaging. (B) Heart-torso model (i), and positions of the electrodes/sensors in the surface of the body (ii). (C) Simulated body-field maps and (D) example single electrode signals, for ECG (i) and MCG (ii). The white arrows show the direction of the electric potential, while the green circled arrows show the direction of the magnetic field, consistent with the right hand rule (C).

A three dimensional (3D) biophysically detailed computational model of the human ventricles was incorporated into a heart-torso model to simulate normal and ischemic conditions (Fig 1). The 3D ventricular anatomical model was previously developed and is segmented into the major distinctive electrically heterogeneous regions [20] (Fig 1A). All of the models incorporated anatomical structures and detailed electrophysiological heterogeneity with cellular electrophysiology being described by the Ten Tusscher et al. single cell model of human ventricular action potentials (TNNP) [21].

#### Single cell model

The TNNP model for human ventricular cells [21] was employed to simulate the action potential (AP) of the myocytes. The formulation of the rapid delayed rectified potassium current (I_Kr_) was replaced by a Markov-chain formulation [20]. The membrane potential can be evaluated by:

$$\frac{dV}{dt}=-\frac{I_{ion}+I_{stim}}{C_{m}}$$ (1)

where V is the voltage across the membrane, t is time, I_ion_ is the total transmembrane current, I_stim_ is the stimulus current applied externally, and C_m_ is the capacitance of the cell. Also, the late component of the sodium current (I_NaL_) was incorporated by adapting the model of I_NaL_ from the O’Hara et al. model [22]. The TNNP model is capable of simulating three types of action potential representing endocardial (ENDO), midcardial (MCELL) and epicardial (EPI) cells (Fig 1A(i)).

#### Tissue Model

The excitation and wave propagation in the tissue was abstracted to be a diffusion-reaction problem, and modelled with the monodomain equation:

$$C_{m}\frac{\partial V}{\partial t}=-\left(I_{ion}+I_{stim}\right)+\nabla \cdot \left(D\nabla V\right)$$ (2)

where **D** is the diffusion tensor describing the conductivities of the tissue along different directions. **D** was set at 0.18 mm^2^/ms along the fibre direction and 0.06 mm^2^/ms across the fibre direction, giving a planar conduction velocity of 71.9 cm/s along fibre direction and 42.5 cm/s across fibre direction. These values are close to the 70 cm/s conduction velocity along the fibre direction found in human ventricles [23].

The activation and wave propagation of excitation was simulated on an anatomically accurate human ventricular geometry reconstructed from DT-MRI imaging data [24]. The derived fibre orientation was incorporated to account for the anisotropy in the material property. In order to simulate the transmural electrical heterogeneity of the ventricle walls, the tissue was segmented into ENDO, EPI and MCELL regions. Specifically, the MCELL region was considered to be isolated islands within the endocardium [25,26]. Apico-basal heterogeneities in the electrophysiological properties of the myocardium were considered by adding gradients to I_Ks_ (slow rectified delayed current) [25,27]. A linear scaling function based on the distance to the base of ventricles was applied to the channel conductance of I_Ks_. As such, the conductance of I_Ks_ in the myocytes in the apex was 2.67-fold larger than that of the basal cells [27]. Empirically determined activation sites across the endo-surface of ventricular walls were used to mimic the Purkinje conduction network, as a detailed structure of such conduction system is not available. These activation sites were validated by reproducing the excitation wave propagation pattern in human ventricles and QRS complex of measured 64-channel ECG [20,28].

#### Modelling ventricular ischemia

**Fig 2:**
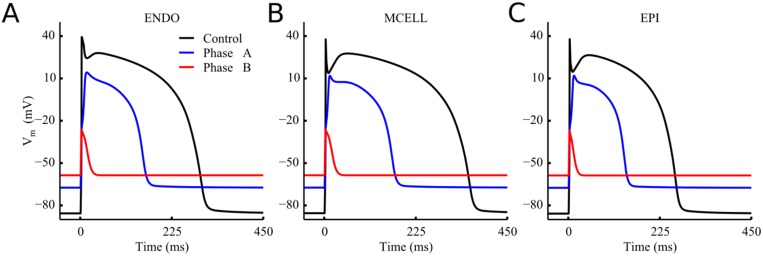
Simulated action potentials under control and ischemic conditions. Simulated action potentials (APs) under control conditions (Black line), phase A (blue line) and phase B (red line) of ischemia. (A) Endocardium. (B) Myocardium cell. (C) Epicardium.

Acute ischemia can be considered in two phases: phase A (first 2–10 minutes post-occlusion) and B (15–45 minutes after coronary occlusion) [29,30]. These two phases were modeled separately by mimicking ischemia induced changes on cardiac electrophysiology [31,32] at 10 and 45 minutes post-occlusion, respectively. In phase A, we considered: (i) hyperkalemia: an increase in extracellular potassium concentration; (ii) acidosis: decrease in the maximum conductivity of sodium and L-type calcium currents, and; (iii) hypoxia: activation of ATP dependent potassium current, I_KATP_ [33,34]. In phase B, changes to sodium-calcium exchanger, sodium-potassium pump and intracellular calcium handling system were introduced in addition to the alterations seen in phase A. The conductivity of tissue was also reduced in phase B but not in phase A [32]. A detailed summary of the changes is given in S1 Table. The resulting changes to ENDO, MCELL and EPI action potentials are shown in Fig 2.

I_KATP_ was modelled using the formula from Kazbanov et al. [33], given by:

$$I_{KATP}=G_{KATP}\cdot f_{KATP}\cdot {\left(\frac{{\left[K\right]}^{+}{}_{0}}{5.4}\right)}^{0.3}\cdot \frac{1.0}{40+3.5\;e^{0.025V}}\left(V-E_{K}\right)$$ (3)

where G_KATP_ is the maximum channel conductance, f_ATP_ the fraction of open gate, [K^+^]_o_ the extracellular potassium concentration, V the membrane potential, E_K_ the Nernst reversal potential for potassium. The parameters of I_KATP_ were kept the same with Kazbanov et al. [33].

To perform a thorough comparison of ischemia induced changes in ECG and MCG, a number of ischemic lesion conditions with a single lesion but in 20 different locations and different sizes were created. To simplify the problem, spherical ischemic regions were used with randomly selected centres throughout the ventricular myocardium. Both small and large lesions (18mm and 27mm in radius respectively) were considered. Similar to previous studies [31,32], the lesions are composed of central zones (CZ) and border zones (BZ), with CZ occupying myocardium within 80% of the lesion radius to the centre. In the BZ, the ischemic parameters were assumed to vary linearly from CZ ischemic parameter to normal.

#### Simulating ECG and MCG

To simulate ECG and MCG, the ventricle models were placed within a previously developed torso model [35], which considers the presence of lungs, liver, stomach, kidneys, blood masses, spinal cord and ribs, each with different electrical conductivities (Fig 1B). The boundary element method (BEM) was used to compute the electric potentials on the surface of the body (Fig 1C), resulting from an applied current density, *J*_*i*_, obtained from the electrical activity of the ventricular tissue-models. Details can be found in previous studies [35,36]. Once the electric potential *ϕ* is known, the magnetic field, ***B***, was obtained by discretizing the volume into *m* homogenous elements and using a BEM of the Biot-Savart law [37,38]:

$$B\left(r\right)=B_{0}-\frac{\mu_{0}}{4\pi }\Sigma_{k=1}^{m}\left(\sigma_{k}^{i}-\sigma_{k}^{e}\right)\int \nabla^{\prime }\phi \left(r^{\prime }\right)\;\times \;\frac{\left(r-r\prime \right)}{{\left|r-r\prime \right|}^{3}}\;dV^{\prime }$$ (4)

where ***B***_**0**_ is the magnetic field produced by the current source *J*_*i*_, $\sigma_{m}^{i}$ and $\sigma_{m}^{e}$ are the inside and outside conductivities of the element *k*, respectively, and ***r*** and ***r’*** are the distance to the observation point and the distance to a volume element *dV’*, respectively. Then, the z-component of the magnetic field was selected in order to compare simulated and experimental data.

Elements of the torso mesh corresponding to the locations of the electrodes and magnetic sensors were selected to simulate 12- and 36- lead ECGs and 36-lead MCG (Fig 1B). The simulated 12- lead and 36-lead ECG and MCG was compared with experimental data.

#### Quantifying the effects of ischemia on ECG and MCG—relative difference

The QRS complex, ST-segment and T-wave were analyzed to compare control with the two different ischemic stages. In order to evaluate the functional effects of ischemia on the spatial distribution pattern of BSP and MCG maps, relative differences in the amplitude of BSP and MCG signals between control and ischemic conditions were calculated during each segment duration of the cardiac excitation rhythm for variant ischemic cases, i.e. the amplitude difference between the control and ischemic cases divided by the amplitude of the control signal at each specific point and multiplied by 100. This is similar to the discriminant index [39], which has been suggested to indicate the capability of each sensor site to distinguish between patients and control [39,40].

## Results

Fig 2 shows the simulated ventricular action potentials of endocardium (ENDO), mid-layer (MCELL) and epicardium (EPI) cells in the control and Phase A & B of ischemic conditions. It was shown that ischemia caused some changes to the profiles of the action potential, including an elevated resting potential (i.e., more positive), reduced amplitude of AP and shortened action potential durations (APDs). Such effects of ischemic condition on cellular AP became more pronounced with time course of ischemia. These simulation results well matched to experimental [30,31] and previous simulation studies [21,33].

### Effects of ischemia on ECG and MCG

**Fig 3:**
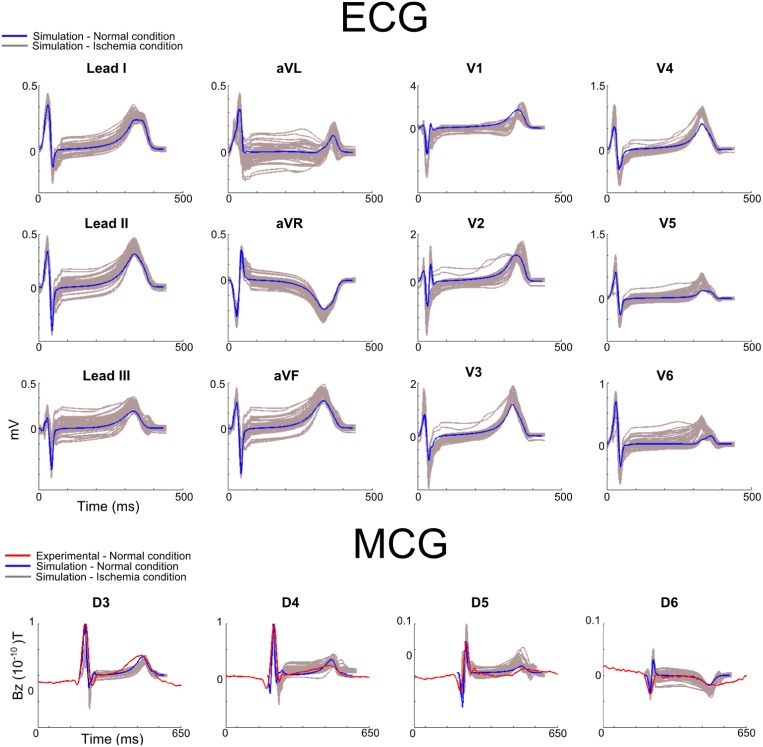
Simulated 12-leads ECG and MCG under control and ischemic conditions. Simulated time courses of 12-lead ECGs (superior), MCGs (from leads D3 to D6; bottom) under control (blue line) and ischemic conditions (Phase A with variant locations; gray lines). Experimental data (red) was included in the MCG for comparison purposes. Simulated 12-lead ECG and MCG were normalized to the maximum amplitude of each lead, and superimposed over experimental recordings from a healthy subject.

First, we investigated the effects of ischemia on 12-lead ECGs, as well as MCGs computed from the leads close to the chest leads for conventional 12-lead ECGs (i.e., leads D3 to D6 in Fig 1). The characteristic features (including polarity of the QRS complex and T-wave, QRS duration and QT intervals) of the time courses of the computed 12-lead ECGs during the control condition were within the range of experimental data of previous studies [1,3,41,42], and also matched our own to experimental recordings (Fig 3-ECG). The simulated MCG time courses from leads D3 to D6 also matched to the experimental data (Fig 3-MCG). The match between simulation data and experimental data validated the developed heart-torso models and algorithms for simulating ECGs and MCGs.

With the validated model, further simulations were performed to theoretically investigate the effects of ischemia on the profiles of 12-leads ECG and MCGs. Results are shown in Fig 3. It was shown that the presence of ischemic condition (Phase A with variant ischemic locations) resulted in noticeable changes to the profiles of the 12-lead ECGs as compared to control (normal) condition (Fig 3-ECG). In simulations, ischemia primarily affected the ST-segment (depending on the ischemic location, it either elevated or depressed the ST-segment) and the T-wave amplitude (depending on the ischemic location, it either increased or decreased the amplitude of the T-wave), and also had an effect but smaller on the QRS complex. These simulation results were consistent with those seen in previous studies [3,31,39,42]. Similar changes were also observed in the simulated MCGs (Fig 3-MCG), which were also consistent with those seen in previous studies [12,13,15,16].

**Fig 4:**
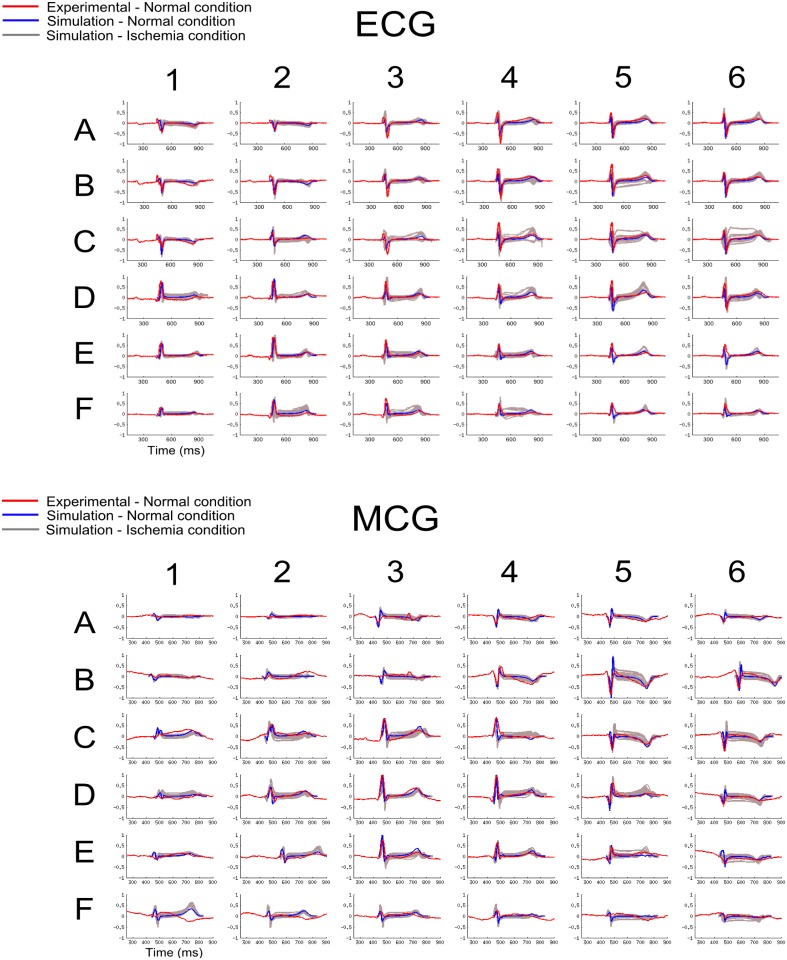
Simulated 36-lead ECG and MCG under control and ischemic conditions. Simulated 36-lead ECG (top panels) and MCG (bottom panels) in control (blue line) and ischemic (grey line) conditions. In control conditions, ECG and MCG were normalized and superimposed with experimental data [18] (red line), simulated data (blue line) during control and ischemic conditions (Phase A with variant locations; grey lines). The numbers and letters represent the electrode/sensor position (Fig 1B-ii). Simulated 36-lead ECG and MCG were normalized to the maximum amplitude of each lead, and superimposed over experimental recordings from a healthy subject.

Fig 4 shows the simulated 36-lead ECG and MCG under control and ischemic conditions. In the control condition, the simulated time courses of QRS complex and T-waves of the 36-lead ECG and MCG showed strong agreement to experimental data [18] (Fig 4) for all of the 36-leads, which further validated the multi-scale models of the ventricle.

In both experimental and simulation data, the polarity of the QRS and T-wave of ECG and MCG signals showed similar spatial distribution patterns. For ECG, the QRS complex was mainly positive in the left-inferior part of the body, negative in the superior right part of the body, and biphasic or flat in intermediary locations (Fig 4-ECG). The T-wave was positive in most of the leads, except for the superior right part of the body. In contrast, for MCG, the polarity of the QRS complex was mainly positive in the right-inferior part of the body, negative in the superior left part of the body, and biphasic or flat in intermediary locations (Fig 4-MCG). The T-wave was positive in most of the leads, except for the superior left part of the body. These simulated spatial distribution patterns of QRS and T-wave of ECG and MCG matched to experimental data.

The effects of ischemia in the 36-leads are also shown in Fig 4. As the 36-leads covered much wider are of the body surface compared to the 12-lead system, simulation data showed more pronounced changes in BSP and MCG signals in some specific regions of the body compared to the 12-lead system. For example, T-wave inversion was seen in some of the 36-leads of both ECG (lead B5) and MCG (lead C4), which was not seen in any of the 12-lead ECGs. Therefore, an analysis of sensitivity regions through the calculation of the relative differences was performed in both ECG and MCG data to quantify the regions with more pronounced changes in signal due to ischemia conditions.

### Sensitivity of ECG and MCG to ischemia

**Fig 5:**
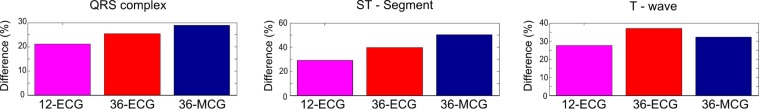
Averaged relative differences of 12- and 36-lead ECG and 36-MCG. Averaged relative differences of the QRS complex, ST- segment and T-wave of the12-lead ECG (pink bar), 36-lead ECG (red bar) and MCG (blue bar) between the ischemic (Phase A & B with variant locations) and control condition.

To analyse the sensitivity of ECG and MCG to ischemia conditions (in both Phase A & B condition and with variant ischemic locations), averaged relative differences were calculated for the 12-lead ECG, 36-lead ECG and 36-lead MCG, for the QRS complex (during the time period of 410–460 ms), ST-segment (550-650ms) and T-wave (700-400ms) time intervals. Results are shown in Fig 5.

For the QRS complex, ischemia induced small differences between ECG and MCG signals. The computed relative differences between ischemia and control conditions were similar for both ECG and MCG, which were 21% for 12-lead ECG, 25% for 36-lead ECG and 29% for the 36-lead MCG respectively (Fig 5, QRS complex).

For the ST-segment, ischemia induced marked changes in both ECG and MCG as compared to control conditions. However, the MCG showed greater sensitivity to ischemia than the ECG. The computed relative differences were 29% for the 12-lead ECG, 40% for 36-lead ECG and 50% for the 36-lead MCG respectively (Fig 5, ST-segment).

For the T-wave, the presence of ischemia produced greater changes in ECG than MCG. The computed relative differences between control and ischemia were 28% for 12-lead ECG, 37% for 36-lead ECG and 32% for 36-lead MCG respectively (Fig 5, T-wave).

#### Regional differences in the sensitivity of ECG and MCG to ischemia

**Fig 6:**
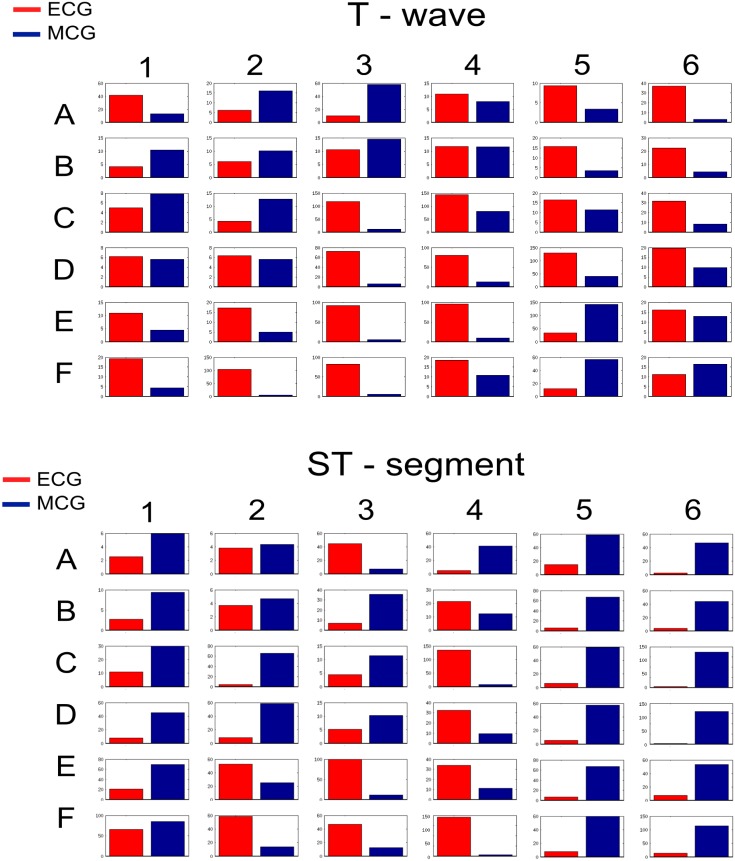
Relative differences of each of the 36-leads ECG and MCG between ischemic and control conditions. Computed relative differences for each of the 36-leads ECG (red) and MCG (blue) with control conditions for the QRS complex, ST-segment, and T-wave.

Next, possible regional differences in the sensitivity of ECG and MCG to ischemia were investigated. As the 36-lead ECG and MCG provide more spatial information of the torso, analysis was conducted in the 36-lead data. Results are presented in Fig 6, showing computed relative differences of ECG and MCG between control and ischemia (during both phases) for each of the 36 leads during the ST-segment and T-wave time intervals. It was shown that the relative difference for both of the ECG and MCG was region-dependent, and was different between the ST-segment and T-wave period. Such differences in the regional dependence between the ST-segment and the T-wave period may account for the discrepancy shown by the averaged relative differences between MCG and ECG regarding their sensitivity to ischemic QRS and T-wave.

**Fig 7:**
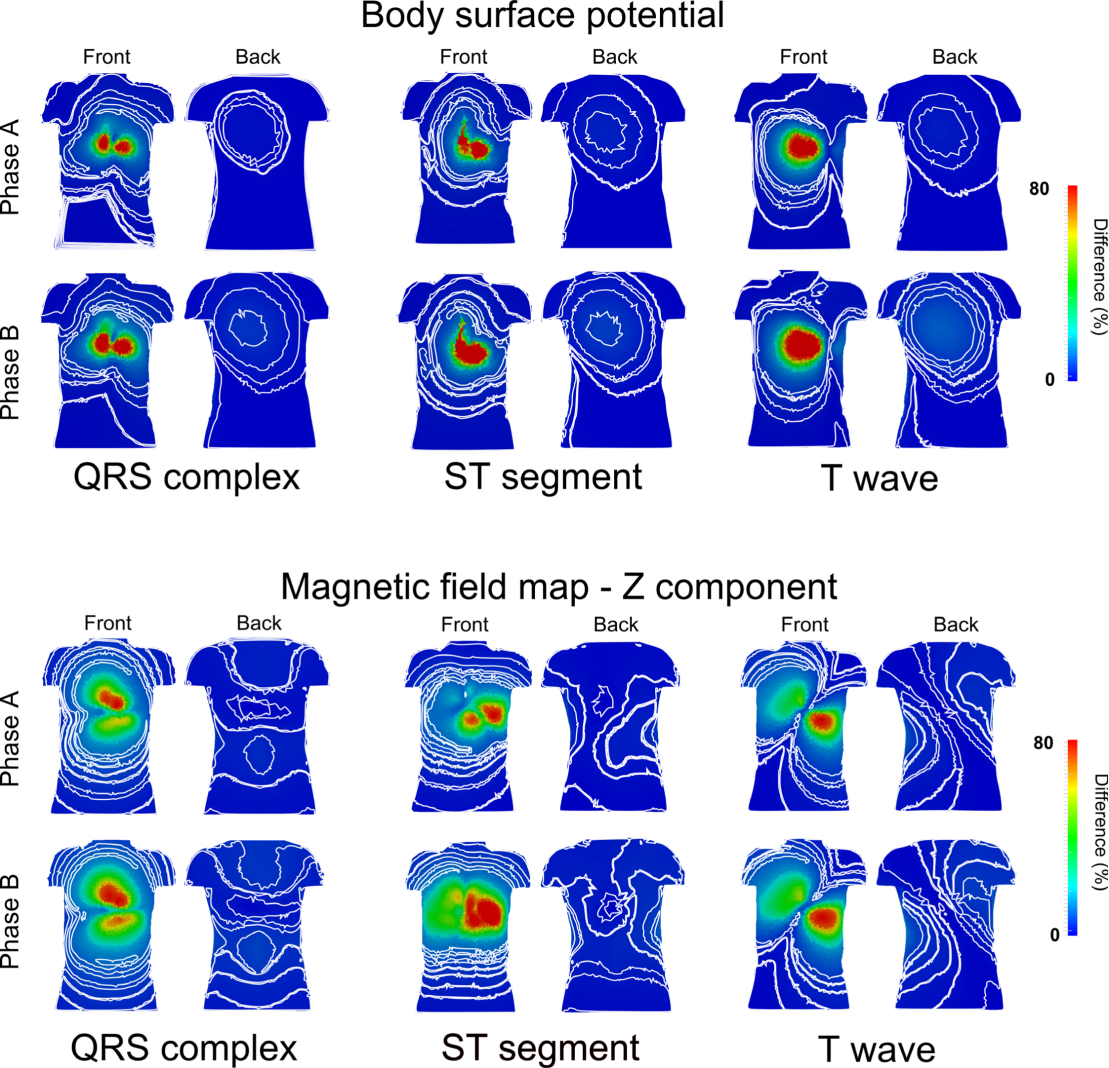
Maps of major differences of BSP and MCG between ischemic and control conditions. Maps of major differences of BSP (top panels) and MCG (bottom panels) between ischemia and control conditions for the QRS complex, ST-segment, and T-wave, during both early (Phase A) and late (Phase B) ischemic phases. The contour lines correspond to small variations in the small difference range, in both frontal (front) and posterior (back) part of the torso.

Fig 7 shows quantified regional relative differences of ECG and MCG for the QRS complex, ST-segment and T-wave in Phase A & B ischemic conditions.

The spatial distribution of the relative difference map of the BSP and MCG between ischemia and control did not show great changes between the two ischemia phases (Fig 7). However, the map of the ST-segment differences showed pronounced changes as compared to control (Fig 7 ST-segment), with more pronounced changes in the MCG maps.

Further investigations were performed to study the effects of variant ischemic locations on profiles of ECG and MCGs. Ischemic region in four sub-sections of the ventricles were considered: the superior left ventricle, inferior left ventricle, superior right ventricle and inferior right ventricle. For each case the relative difference between control and ischemia was computed.

**Fig 8:**
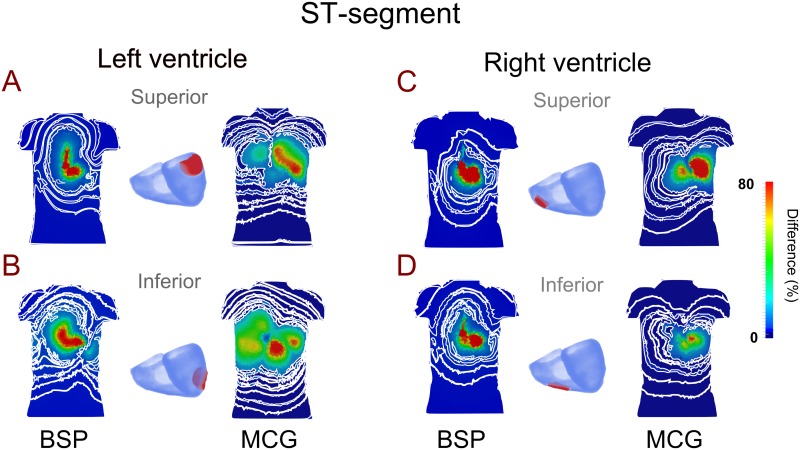
Maps of the major difference of BSP and MCG between control and localized ischemic condition. Maps of the major difference of BSP and MCG between control and varied localized ischemic condition during the ST-segment. Ischemic region was considered in four different ventricular locations (labeled in red): Superior left ventricle (A), inferior left ventricle (B), superior right ventricle (C), inferior right ventricle (D). The contour lines correspond to small variations in the small difference range.

Fig 8 presents the relative difference map of ECG and MCG signals, showing a correlation between the ischemic region of the heart and the torso region with maximal relative changes in the ST-segment of MCG, but not ECG. Our simulation data showed that an ischemic region located in the superior left ventricle produced a maximal relative change of MCG signals in the superior part of the torso (Fig 8A). An ischemic region located in the inferior part of the left ventricle produced a maximal change of MCG signals in the superior and inferior right of the torso (Fig 8B). Similarly, an ischemic region located in the superior part of the right ventricle produced a maximal relative change of MCG signals in the superior-left part of the torso (Fig 8C), and an ischemic region in the inferior right ventricle produced a maximal relative change of MCG signals in the left-mid part of the torso (Fig 8D). However, there was no such correlation observed from the ECG map.

## Discussion

### Major contribution

It remains controversial whether or not MCG signals can provide useful extra information for more effective diagnose and characterization of cardiac diseases, mainly the one asymptomatic to the ECG [23,33,43]. Previous studies have shown that both ECG and MCG can produce similar results during specific silent ischemia [43], while other studies have suggested that circular vortex currents can be detected by MCG but not by ECG [16,44,45]. Moreover, the ischemic injury might increase the tangential current flow through the ventricular tissue, producing the repolarization abnormalities, which are detected differently by MCG and ECG [17,44,45]. In this study, by using a well validated biophysically detailed computer model of human ventricles-torso, we computationally investigated the different features of 12-, 36-lead ECG and MCG, BSP and MCG maps during normal and variant ischemic conditions. We also investigated the regional dependence of the measured relative difference and how the area with maximal relative difference on the body surface varied due to altered stage and location of the ischemic region.

Our major findings are: (i) both the 36-lead ECG and MCG showed greater averaged relative difference in the QRS complex, T-wave and ST-segment than the 12-lead ECG, indicating the advantages of implementing multi-lead ECG/MCG systems than the conventional 12-lead ECG in diagnosing the ischemic condition; (ii) ECG and MCG showed a different sensitivity to ischemia in producing changes in the T-wave and ST-segment. Our results showed that the 36-leads ECG was more sensitive than the 36-leads MCG in detecting changes in the T-wave by producing a greater averaged relative difference in the T-wave. However, for detecting changes in the ST-segment the MCG showed greater sensitivity by producing a greater relative difference; (iii) both ECG and MCG showed regional-dependent changes to ischemic condition on the body surface of the torso, but with MCG showing a stronger correlation between ischemic region in the heart and the maximal difference map on the body surface. Such difference in the sensitivity between ECG and MCG may be due to the different effects produced by the ischemia to the AP (an elevation in the resting potential, reduced amplitude of AP and shorter APDs), which mainly affects the repolarization propagation that is associated with ST-segment and T-wave. Such a cellular effect maps to electrical wave propagation, producing different tangential and normal currents leading to altered electrical and magnetic fields; and (iv) the correlation between ischemic region in the heart and the maximal relative differences of MCG during QRS complex, ST-segment and T-wave provides a theoretical basis for non-invasively diagnosing ischemic region.

### Limitations

The limitations of used cellular models for human ventricular action potentials have been discussed in previous studies [20,21], and such limitations were inherited in the present study. In addition, the torso model lacks considerations of some tissue types or organs (such as muscles, fat tissue) that may affect the amplitude of simulated surface potentials. Nevertheless, the absence of those tissues does not have a significant effect on the polarity of the ventricular-waves [35,46], and produces less effect in the MCG measurements [12,44].

In the present study, by comparing the regional-dependence of the averaged relative difference map between control and ischemic ECG/MCG signals, we were able to show a strong correlation between the ischemic region in the heart and maximal relative difference in the MCG. However, it should be pointed out that for both experimentally recorded and simulated MCG its amplitude decreases significantly in the areas far from the heart position, i.e., back of the torso or close to the limbs. This causes smaller changes in the MCG compared to the ECG in these areas of the body, which may limit the accuracy to detect ischemic region by using a whole body MCG map. A possible solution to overcome such technique limitations is to use combined high spatial resolution BSP and MCG maps, which provide a feasible tool to diagnose cardiac ischemic in clinical environments.

Note that there are still some controversies regarding whether or not MCG can provide some extra information than ECG in dragonizing cardiac diseases. For better analysis MCG information, MCG maps were reconstructed, with some studies based on the MCG maps morphology [13,18,44,45,47], and others on the amplitude [43,18], the polarity of the signal [13], or current-arrow maps reconstructed by subtraction of waveforms [48,49]. Few studies have tried to standardize the MCG polarity maps [47], or presented full detailed ECG and MCG maps together with the axis system and electrode positions. All of these detailed are needed for future studies in order to perform a better standardization and comparison between simulation and experimentation. The aim of this study was not to find a standard MCG maps, but actually to quantitatively compare the averaged differences in the sensitivity in locating variant different ischemic regions in the ventricles between using 12-, 36- leads ECG and MCG signals. To our best knowledge, this is the first study to show regional-dependent changes of maximal MCG difference map to ischemic conditions, which was not found in the ECG.

## Conclusion

Computer modelling provides a useful tool to compare the electric and magnetic field produced by the electrical activity of the heart during normal and ischemic conditions, which is a challenging task in clinical settings. Using biophysically detailed models of human ventricles and torso, we have compared the sensitivity regions of the ECG and MCG in response to ischemic conditions. Our results suggest that the 12-lead ECG is less effective to provide diagnosis of the ischemia, whereas the 36-lead ECG and in particular MCG offer advantages in the identification of ischemic conditions. By comparing the relative differences in the BSP and MCG maps, our results shows that MCG has greater sensitivity than ECG in response to ischemia, which may provide an alternative method for the diagnosis of ischemia.

## Supporting Information

S1 Table Table of ischemia induced changes to ventricular electrophysiology. Summary of ischemia induced changes to the electrophysiological properties of the human ventricles.
(DOCX)
