# Supplementary material for: Comparison of Electric- and Magnetic-Cardiograms Produced by Myocardial Ischemia in Models of the Human Ventricle and Torso
Source: PLoS One. 2016 Aug 24;11(8):e0160999. doi: 10.1371/journal.pone.0160999 (PMC4996509; doi:10.1371/journal.pone.0160999)
Supplement: S1 Table — Summary of ischemia induced changes to the electrophysiological properties of the human ventricles. (DOCX) [file pone.0160999.s001.docx]

**Suporting information Table S1**

Comparison of Electric- and Magnetic-cardiograms Produced by Myocardial Ischemia in Models of the Human Ventricle and Torso

Erick A Perez Alday^1^, Haibo Ni^1^, Chen Zhang^2^, Michael A Colman^3^, Zizhao Gan^2^, Henggui Zhang^1*^

*^1^Biological Physics Group, Department of Physics and Astronomy, University of Manchester, Manchester, United Kingdom,*

*^1^Biological Physics Group, Department of Physics and Astronomy, University of Manchester, Manchester, United Kingdom,*

*^3^Applied superconductivity Research Center, School of Physics, Peking University, Beijing, China.*

*Correspondence: henggui.zhang@manchester.ac.uk

Table 1. Summary of ischemia induced changes to the electrophysiological properties of the human ventricles.

| Parameters |  | Control | Phase A | Phase B |
| --- | --- | --- | --- | --- |
| [K+]o |  | 5.4 | 10.0 | 15.0 |
| fATP |  | 0 | 0.55% | 0.55% |
| SF_GNa |  | 1.0 | 0.8 | 0.5 |
| SF_GCaL |  | 1.0 | 0.8 | 0.5 |
| SF_INaCa |  | 1.0 | 1.0 | 0.2 |
| SF_INaK |  | 1.0 | 1.0 | 0.3 |
| SF_JRyR |  | 1.0 | 1.0 | 0.05 |
| SF_Jup |  | 1.0 | 1.0 | 0.9 |
| GNaL |  | 0.0065 ms/μF | 0.065 ms/μF | 0.065 ms/μF |
| SF_Diffusion |  | 1.0 | 1.0 | 0.5 |
